# The antibiotic strategies during percutaneous nephrolithotomy in China revealed the gap between the reality and the urological guidelines

**DOI:** 10.1186/s12894-022-01092-7

**Published:** 2022-08-30

**Authors:** Shike Zhang, Gonghui Li, Ludong Qiao, Dehui Lai, Zhican He, Lingyue An, Peng Xu, Hans-Göran Tiselius, Guohua Zeng, Junhua Zheng, Wenqi Wu

**Affiliations:** 1 Department of Urology, The Second Affiliated Hospital of Guangzhou Medical University, 250 Changgang Road, Haizhu District, Guangzhou, 510260 Guangdong China; 2 Guangdong Key Laboratory of Urology, Guangzhou Medical University, Guangzhou, China; 3 Department of Urology, Sir Run-Run Shaw Hospital College of Medicine, Zhejiang University, Hangzhou, China; 4 Department of Urology, Beijing Tongren Hospital, Capital Medical University, Beijing, China; 5 Department of Urology, The Fifth Affiliated Hospital of Guangzhou Medical University, Guangzhou, China; 6 Department of Urology, The First Affiliated Hospital of Guangzhou Medical University, 1 Kangda Road, Haizhu District, Guangzhou, 510230 Guangdong China; 7 Division of Urology, Department of Clinical Science, Intervention and Technology (CLINTEC), Karolinska Institutet, Stockholm, Sweden; 8 Department of Urology, Shanghai General Hospital, Shanghai Jiao Tong University School of Medicine, Shanghai, China; 9 Department of Urology, Shanghai General Hospital, The First People’s Hospital Affiliated to Shanghai Jiaotong University, Haining Road #100, Hongkou District, Shanghai, 200080 China

**Keywords:** Percutaneous nephrolithotomy, Antibiotic, Current practice, Guideline, Survey

## Abstract

**Background:**

Correct perioperative antibiotic strategies are crucial to prevent postoperative infections during percutaneous nephrolithotomy (PCNL). We aimed to compare the realistic antibiotic strategies applied in China with current urological guidelines.

**Methods:**

Between April and May 2020, urologists from China were invited to finish an online cross-sectional survey. The questionnaire was designed according to the current urological guidelines and literatures.

**Results:**

3393 completed responses were received. 61.1% (2073/3393) respondents had urological experience of more than 10 years. 72.4% urologists chose multiple-dose antibiotics for patients with both negative urine culture (UC-) and negative urine microscopy (UM-) preoperatively. Respondents in central China (OR = 1.518; 95% CI 1.102–2.092; *P* = 0.011), east China (OR = 1.528; 95% CI 1.179–1.979; *P* = 0.001) and northeast China (OR = 1.904; 95% CI 1.298–2.792; *P* = 0.001) were more likely to prescribe multiple-dose antibiotic for UC-UM- patients. Notably, the respondents who finished PCNL exceeded 100 cases per year were in favor of single-dose administration (OR = 0.674; 95% CI 0.519–0.875; *P* = 0.003). There are only 8.3% urologists chose single-dose antibiotic for UC-UM+ patients, whereas 65.5% administered antibiotics for 1–3 days. Meanwhile, for UC+ patients, 59.0% of the urologists applied antibiotics shorter than 1 week, and only 26.3% of the urologists carried out routine re-examination of UC. Moreover, postoperative antibiotics were frequently prescribed for 3–6 days (1815; 53.5%). Finally, although 88.2% urologists considered stone culture important for management of postoperative antibiotics as the guideline recommended, only 18.5% performed it routinely.

**Conclusions:**

The antibiotic strategies are different between current practice in China and the urological guidelines. The dissimilarities suggested that further studies should be conducted to investigate the reasons of the differences and standardize the application of antibiotics.

## Introduction

Although percutaneous nephrolithotomy (PCNL) is widely and successfully used in the management of patients with upper urinary tract stones [1], infection complications, especially urosepsis is still catastrophic and associated with significant morbidity [2]. According to the guidelines of the Chinese Urological Association (CUA), the American Urological Association (AUA) and the European Association of Urology (EAU), the application of antibiotics for PCNL patients perioperatively is a well-recognized strategy to prevent postoperative infections [3, 4]. However, guideline’s recommendations cannot be given in every detail. Single-dose prophylactic antibiotic before PCNL for patients with negative urine culture (UC−) was recommend in both EAU and AUA guidelines [4, 5], but the CUA guideline additionally recommend antibiotic treatment for 3–5 days in UC− patients with positive urine microscopy (UM+) [3], while the Japanese guideline suggested that patients with stones ≥ 2 cm or hydronephrosis should receive 1 week of antibiotic treatment. Moreover, despite emphasizing that urinary tract infections (UTIs) should be controlled primarily based on UC and the bacteria sensitivity patterns whenever stone removal is planned [3–5], there are no clear strategies regarding doses or duration of antibiotic treatment. The strategies for post-PCNL antibiotics are also insufficiently clear. Therefore, the urologists potentially use antibiotics in several different practice patterns in PCNL [7, 8].

Until now, there is limited information revealing the current practice of antibiotics in PCNL in China. Therefore, Investigation on how antibiotics actually used in PCNL will provide valuable information on the most appropriate strategies and hopefully reduce nonstandard use of antibiotics. The aim of this study was to assess the current status of antibiotic practice among Chinese PCNL patients, and the differences with current recommendations of urological guidelines.

## Materials and methods

### Study design and participants

An online questionnaire was conducted under the advices of the members from the society for inflammation and infection of the Chinese Urological Association (SII-CUA). The questionnaire was then distributed to Chinese urologists via different online WeChat groups and Wenjuanxing survey platform. The study stayed open from April 1 to May 31, 2020. Chinese urologists in registration were invited to engage in this study.

### Study instrument

To ensure sufficient quality of the study, the questionnaire development followed a three-phase process comprising a literature review for item generation, an expert panel review, and a pre-test. Initially, 30 questions were drawn from a literature review of previous studies exploring antibiotic strategies in PCNL. All questions were grouped in discrete categories, including demographic details and occupational features, attitude to laboratory tests of UTIs, cognition of risk factors and symptoms of post-PCNL infections (e.g., preoperative history of fever), and antibiotic behaviors in PCNL. These questions were subsequently submitted to experts in SII-CUA and evaluated. Extra items were integrated, others were deleted and valuable questions were added after discussion and pre-test outcome.

Urologists were asked about their preferences in daily routine rather than what they consider the most correct answers. Skip logic was introduced within the questionnaire framework to decrease the answer time, but responses with the answer time shorter than 2 min were considered invalid. Data were collected electronically and finally anonymously tabulated.

### Statistical analysis

Data analysis was carried out using SPSS (Version 24, IBM, New York, USA). Descriptive statistics including demographic and professional characteristics of respondents were reported by numbers and frequency. Logistic regression analysis was used to introduce odds ratios (OR) and 95% confidence intervals (95% CI) to determine independent factors associated with multiple-dose pre-PCNL antibiotics. Results with two-sided *p* < 0.05 were considered statistically significant.

## Results

A total of 3393 completed questionnaires were validated from 3480 original responses, with an average answer time of 7.4 min. 61.1% (2073/3393) of the respondents had more than 10 years of urological experience, and 69.1% (2343/3393) worked in tertiary hospitals. The annual volume of PCNL procedures was more than 50 cases for 42.7% of the respondents (Table 1).

**Table 1:** Basic characteristics of the respondents (N = 3393)

Characteristics of the respondents	Survey participants	
	N	Frequency (%)
Years of experience (practice as urologist)		
< 1 year	112	3.3
1–3 years	219	6.5
4**–**5 years	273	8.0
6–10 years	716	21.1
> 10 years	2073	61.1
Surgical experience of PCNL per year		
< 10 cases	563	16.6
10–50 cases	1381	40.7
51–100 cases	778	22.9
> 100 cases	671	19.8
Grade of attaching medical unit		
Tertiary hospital	2343	69.1
Non-tertiary hospital	1050	30.9
University hospital		
Yes	1640	48.3
No	1753	51.7
Geographical area		
East China	777	22.9
North China	466	13.7
Central China	339	10.0
South China	588	17.3
Northeast China	223	6.6
Northwest China	369	10.9
Southwest China	631	18.6

### The practice of pre-PCNL antibiotics

Preoperatively, UM was approved for diagnosis of UTIs by 3385 (99.8%) respondents and 3065 urologists (90.3%) routinely performed UC for their patients. There were 3345 (98.6%) urologists who reported prescription of pre-PCNL antibiotics for patients with negative UC and UM (UC-UM−). Most urologists (2221; 65.5%) selected a treatment course of 1–3 days, while 27.6% (936/3393) used a single-dose administration (Table 2). Notably, the respondents who finished PCNL exceeded 100 cases per year were in favor of single-dose administration (OR = 0.674; 95% CI 0.519–0.875; *P* = 0.003). Meanwhile, respondents in central China (OR = 1.518; 95% CI 1.102–2.092; *P* = 0.011), east China (OR = 1.528; 95% CI 1.179–1.979; *P* = 0.001) and northeast China (OR = 1.904; 95% CI 1.298–2.792; *P* = 0.001) were more likely to prescribe multiple-dose of pre-PCNL antibiotic for UC-UM- patients (Table 3). Cephalosporin was used as the most common type of antibiotic (2323; 69.4%), followed by quinolones (561; 16.8%) and beta-lactamase inhibitors (227; 6.8%) for UC− patients.

**Table 2:** Current pre-PCNL antibiotic regimens reported by Chinese urologists

Pre-PCNL antibiotic treatment	Survey participants	
	N	Frequency (%)
Duration of antibiotic treatment for UC-UM- patients		
None	48	1.4
Single dose before anesthesia induction	936	27.6
1–3 days	2221	65.5
4–6 days	140	4.1
** ≥ **7 days	48	1.4
Types of antibiotics for UC-UM- patients (N = 3345)		
Aminoglycosides	31	0.9
Cephalosporins	2323	69.4
Quinolones	561	16.8
Penicillins/Semisynthetic Penicillins	185	5.5
Carbapenems	18	0.5
Beta-lactamase inhibitors	227	6.8
Duration of antibiotic treatment for UC-UM+ patients		
None	9	0.3
Single dose before anesthesia induction	282	8.3
1–3 days	1849	54.5
4–6 days	326	9.6
** ≥ **7 days	78	2.3
Until infection alleviated	631	18.6
Until leucocytes and/or nitrites in UM turn negative	218	6.4
Types of antibiotics for UC-UM+ patients (N = 3384)		
Aminoglycosides	14	0.4
Cephalosporins	2243	66.3
Quinolones	672	19.9
Penicillins/Semisynthetic Penicillins	202	6.0
Carbapenems	33	1.0
Beta-lactamase inhibitors	220	6.5
Recheck UM for UC-UM+ patients after antibiotic treatment (N = 3384)		
Yes	3228	95.4
No	156	4.6
Duration of antibiotic treatment for UC+ patients		
1–3 days	840	24.8
4–6 days	1161	34.2
7–14 days	547	16.1
> 14 days	22	0.6
Until UC turn negative	823	24.3
The proportion of UC+ patients rechecking UC after antibiotic treatment		
< 25%	1043	30.7
25–50%	565	16.7
51–75%	314	9.3
> 75%	579	17.1
Almost 100%	892	26.3
Administration route		
Intravenous administration	3014	88.8
Oral administration	379	11.2

**Table 3:** Factors associated with multiple-dose of pre-PCNL antibiotics for UC-UM- patients in logistic regression analysis (N = 3345)

	N	Multiple dose		Bivariate analysis			Multivariable analysis		
		n	%	OR	95% CI	P	OR	95% CI	*P*
Grade of affiliation to medical unit						0.908			
Non-tertiary hospital	1035	744	71.9	1					
Tertiary hospital	2310	1665	72.1	1.010	0.858–1.189				
University hospital						0.505			
No	1732	1256	72.5	1					
Yes	1613	1153	71.5	0.950	0.817–1.105				
Geographical area						**0.004**			**0.004**
North China	455	308	67.7	1					
Southwest China	626	433	69.2	1.071	0.826–1.388	0.606	1.159	0.889–1.510	0.276
South China	581	408	70.2	1.126	0.864–1.467	0.382	1.242	0.945–1.632	0.120
Northwest China	361	258	71.5	1.195	0.884–1.616	0.245	1.235	0.912–1.671	0.172
Central China	336	250	74.4	1.387	1.013–1.899	**0.041**	1.518	1.102–2.092	**0.011**
East China	765	576	75.3	1.455	1.126–1.879	**0.004**	1.528	1.179–1.979	**0.001**
Northeast China	221	176	79.6	1.867	1.274–2.735	**0.001**	1.904	1.298–2.792	**0.001**
Years of experience (practice as urologist)						0.657			
< 1 year	109	79	72.5	1					
1–3 years	215	153	71.2	0.937	0.561–1.566	0.804			
4**–**5 years	267	187	70.0	0.888	0.541–1.457	0.637			
6–10 years	705	523	74.2	1.091	0.694–1.716	0.706			
> 10 years	2049	1467	71.6	0.957	0.622–1.473	0.842			
Surgical experience of PCNL per year						**0.017**			**0.016**
< 10 cases	553	415	75.0	1					
10–50 cases	1367	1001	73.2	0.909	0.725–1.141	0.412	0.882	0.701–1.109	0.283
51–100 cases	763	546	71.6	0.837	0.652–1.073	0.160	0.800	0.619–1.032	0.086
> 100 cases	662	447	67.5	0.691	0.537–0.890	**0.004**	0.674	0.519–0.875	**0.003**

The values in bold represent P＜0.05

Moreover, 99.7% (3384/3393) of the respondents prescribed pre-PCNL antibiotics for UC-UM+ patients, and 54.5% (1849/3393) applied for 1–3 days. 66.3% (2243/3384) urologists chose cephalosporins (Table 2). Single-dose administration was used only by 8.3% (282/3393) of the urologists. 95.4% (3228/3384) urologists re-examined UM after treatment, but only 25.0% (849/3384) would adjust the antibiotic regimen accordingly (Table 2).

For UC+ patients, antibiotics according to the microbiological sensitivity profile were most frequently prescribed in a course of < 7 days (2001; 59.0%). 16.7% (547/3393) of the urologists would choose a course of ≥ 1 week, whereas 24.3% (823/3393) favored continuous treatments until UC-. Meanwhile, only 26.3% (892/3393) urologists ordered a routine re-examination of UC.

Besides applying antibiotics according to UTIs, 91.7% (3112/3393) of the respondents prolonged the course of antimicrobial treatment in case of history of fever preoperatively. Other reasons for prolonged treatment also included image manifestations of perirenal inflammatory exudation (2994; 88.2%), concomitant diabetes (2898; 85.4%), large stone burden (2426; 71.5%) and long-term indwelling catheter (2411; 71.1%). In comparison, patients with concomitant hydronephrosis (1626; 47.9%) and a history of flank pain (1063; 31.3%) received less attention.

### The practice of post-PCNL antibiotics

Postoperatively, there were 0.8% (26/3393) of the respondents who did not prescribe antibiotics routinely (Table 4). 53.5% of the respondents (1815/3393) reported a course of 3–6 days, and 23.3% (791/3393) would use antibiotics less than 48 h. There are 9.3% (317/3393) of the respondents administrated antibiotics until removal of the nephrostomy tubes. A majority of respondents upgraded the antibiotic level for patients with pyuria (3091; 91.1%), postoperative fever > 38.0℃ (2865; 84.4%), chills (2817; 83.0%), hypotension (blood pressure < 90/60 mmHg; 2514; 74.1%) and leukopenia (WBC count < 4000 cells/ul; 2343; 69.1%), whereas long operation time (> 2 h; 1985; 58.5%), leukocytosis (WBC count > 12,000 cells/ml; 1905; 56.1%), and tachycardia (heart rate > 90/min; 1791; 52.8%) were more likely to be neglected. When postoperative urosepsis is suspected, a carbapenem agent dominated the choice for antibiotic escalation in absence of microbiological sensitivity profile (1816; 53.5%), followed by high-grade cephalosporins (870; 25.6%) or beta-lactamase inhibitors (634; 18.7%). Moreover, even though most urologists (2994; 88.2%) reported to adjust of postoperative medication according to stone culture (SC), only 18.5% (627/3393) routinely carried out SC.

**Table 4:** Current practice in post-PCNL antibiotic treatment reported by Chinese urologists (N = 3393)

Post-PCNL antibiotic treatment	Survey participants	
	N	Frequency (%)
Duration of antibiotic treatment for patients without postoperative infections		
None	26	0.8
≤ 24 h	268	7.9
≤ 48 h	791	23.3
Postoperatively 3–6 days	1815	53.5
Postoperatively 1–2 weeks	176	5.2
Until removal of nephrostomy tubes	317	9.3
Types of antibiotics in post-PCNL empirical treatment		
Quinolones	33	1.0
The third/fourth-generation cephalosporins	870	25.6
Carbapenems	1816	53.5
Beta-lactamase inhibitors	634	18.7
Others*	40	1.2
Adjust post-PCNL antibiotic regimen according to SC result		
Yes	2994	88.2
No	399	11.8
Prescribe SC for PCNL patients routinely		
Yes	627	18.5
No	2766	81.5

*Others includes Penicillins, Aminoglycosides, and Peptide antibiotics

Further analysis was performed to investigated whether factors such as the affiliation and academic status of hospital affecting the choice of antibiotic strategies. The respondents were included in “matched group” when their practices followed the EAU, AUA and CUA guidelines, while other respondents were included in “unmatched group”. The characteristics of respondents in matched group and unmatched group were showed in Table 5. Urologist’s choices of antibiotic regimen during PCNL were influenced by surgical experience and hospital grade. Compared with the urologists with annual PCNL < 10 cases, the urologists with annual PCNL > 100 cases were more likely to follow the guideline recommendations (28.2% vs. 19.2, *P* = 0.002, Bonferroni correction). Meanwhile, urologists from tertiary hospital were also more in favor of guideline recommendations (77.2% vs. 68.5%, *P* = 0.001).

**Table 5:** The characteristics of respondents in matched group and unmatched group

	Matched group (n = 202)	Unmatched group (n = 3191)	*P*
Years of experience (practice as urologist), n (%)			0.714
< 1 year	4 (2.0)	108 (3.4)	
1–3 years	15 (7.4)	204 (6.4)	
4**–**5 years	19 (9.4)	254 (8.0)	
6–10 years	40 (19.8)	676 (21.2)	
> 10 years	124 (61.4)	1949 (61.1)	
Surgical experience of PCNL per year, n (%)			**0.012**
< 10 cases	25 (12.4)	538 (16.9)	
10–50 cases	74 (36.6)	1307 (41.0)	
51–100 cases	46 (22.8)	732 (22.9)	
> 100 cases	57 (28.2)	614 (19.2)	
Grade of attaching medical unit, n (%)			**0.001**
Tertiary hospital	156 (77.2)	2187 (68.5)	
Non-tertiary hospital	46 (22.8)	1004 (31.5)	
University hospital, n (%)			0.285
Yes	105 (52.0)	1535 (48.1)	
No	97 (48.0)	1656 (51.9)	
Geographical area, n (%)			0.292
East China	10 (5.0)	213 (6.7)	
North China	29 (14.4)	437 (13.7)	
Central China	42 (20.8)	735 (23.0)	
South China	30 (14.9)	558 (17.5)	
Northeast China	19 (9.4)	320 (10.0)	
Northwest China	21 (10.4)	348 (10.9)	
Southwest China	51 (25.2)	580 (18.2)	

The values in bold represent P＜0.05

## Discussions

Prevention and treatment of infection is a crucial part of the care of patients in the perioperative period of PCNL [4, 5]. The present study firstly reveals the current practice of perioperative antibiotics in patients treated with PCNL among Chinese urologists.

Preoperative use of prophylactic antibiotics is supposed to reduce the potential bacterial load in urine [9]. Single-dose administration was recommended in AUA and EAU guidelines [4, 5], as well as a joint guideline of the EAU Section of Urolithiasis and International Alliance of Urolithiasis [10]. In a recent multicenter RCT, Chew et al. also concluded that a preoperative single dose of prophylactic antibiotics was sufficient in UC− patients, compared with courses of more than 7 days [11]. It is worth noting that the positive leukocyte reaction combined with positive nitrite in preoperative UM was an early predictor of post-PCNL urosepsis [12]. He et al. reported that UC-UM+ patients should be treated with extended period of antibiotic administration [13]. In our study, most urologists prescribed multiple-dose antibiotics of 1–3 days of cephalosporins for UC− patients, irrespective of the UM results. However, recent studies showed that multi-dose antibiotics could not reduce the rate of postoperative infections in UC-UM+ patients [14, 15], even though the frequency of positive urine nitrite and leukocyte reaction could be decreased significantly following preoperative antibiotic therapy [14]. Therefore, the benefits of multi-dose antibiotics before PCNL for UC-UM+ patients are controversial. It was a common strategy to identify the risk factors of infections and start antimicrobial treatment as early as possible [4, 5]. A prolonged duration of pre-PCNL antibiotics might of value in patients with high risk factors for postoperative infections, such as indwelling urinary drainage tubes, diabetes, and hydronephrosis [3, 6, 16].

Preoperative routine UC is of great importance for recognizing UTIs in stone patients and as a guide for selecting perioperative antibiotics. Despite the recommendations for the duration of perioperative antibiotics is lack in AUA and EAU guidelines for UC+ patients, recent studies showed that those patients should be treated at least 7 days preoperatively with appropriate antibiotics [15–18]. Similarly, the CUA guideline recommends a 1–2 week-course of antibiotics for UC+ patients [3]. However, 59% of the urologists in this study chose less than one week.

An antibiotic course less than 24 h was recommended for healthy individuals postoperatively in AUA guidelines [5, 19]. In this study, 53.5% of the urologists reported a relatively long protocol of postoperative antibiotics (3–6 days). However, long-term antibiotics after PCNL have not been found beneficial to prevent infections [2]. Prolonged antibiotic treatment after PCNL could be attributed to the consideration of high risk of infections. Notably, we found that the hospital grade, geographical location and surgical experience making a difference in urologist’s antibiotic practice patterns in PCNL. The insufficient awareness of guideline recommendations among Chinese urologists might be an important reason for the discretionary use of antibiotics.

In PCNL, a stone specimen collection was strongly recommended by EAU and AUA for SC after lithotripsy in order to guide selection of postoperative antibiotics [4, 5]. In this study, respondents seemed to be aware enough of the significance in SC (88.2%), but only 18.5% of the urologists carried out SC in clinical routines. Promoting SC among Chinese urologists apparently is urgently needed, as a positive SC is closely associated with post-PCNL infections even when UC− [17, 20].

The types of antibiotics for PCNL could also have an impact on the incidence of post-PCNL infections. In our investigation, the cephalosporin agents were most frequently used by Chinese urologists before PCNL, followed by quinolones. However, many studies from different regions of China have showed that the majority of uropathogens isolated from urine or stone in patients with urinary stones were in high resistance to the cephalosporin agents (e.g., cefuroxime and ceftriaxone) and quinolones (e.g., ciprofloxacin and levofloxacin) [21–24]. The EAU guideline exactly suggest that antibiotics should be selected based on local bacterial profiles and drug sensitivity. Therefore, it is recommended that every stone patient should be given UC before PCNL and SC postoperatively, since it was reported that taking antibiotics based on SC results could reduce the incidence of post-PCNL infections [15].

The survey was limited by several factors. Firstly, we cannot estimate the response rate, since we failed to figure out how many subjects received and opened the link of investigation. Secondly, the association between antibiotic behavior and postoperative infections was not explored. However, the survey revealed the current antibiotic strategies of PCNL in China are different with the urological guidelines.

In conclusion, compared with the guidelines this study showed that UC− patients are more likely to receive pre-PCNL antibiotics for 1–3 days irrespective of the UM results, while those with UC+ frequently are given an course < 7 days. For patients without post-PCNL infection, the duration of postoperative antibiotics was undoubtedly longer than that in the guideline recommendations. Moreover, there was a low rate of SC, which needs further attention in the clinical practice. In our opinion, single-dose antibiotic is enough for UC-UM- patients. The antibiotic strategies for the UC− patients but with high risk factors of infection, such as staghorn stone, diabetes, UM+ or existing indwelling urinary drainage tube should be studied further. The selection of antibiotic for UC− patients should be based on the local bacterial spectrum. For UC+ patients, it is necessary to prescribe antibiotics according to the drug sensitivity test and it is better to use them for more than 1 week. Meanwhile, SC is helpful to guide the postoperative antibiotic regimen. Therefore, it is suggested to take measures such as strengthening the Continuing Medical Education for graduated urologists to standardize the application of antibiotics.

## Data Availability

The datasets generated and analysed during the current study are not publicly available due another ongoing analysis but are available from the corresponding author on reasonable request.

## Abbreviations

PCNL: Percutaneous nephrolithotomy
CUA: The Chinese Urological Association
AUA: The American Urological Association
EAU: The European Association of Urology
UC: Urine culture
UM: Urine microscopy
SC: Stone culture
SII-CUA: The society for inflammation and infection of the Chinese Urological Association
UTIs: Urinary tract infections
